# Untargeted lipidomics reveals progression of early Alzheimer’s disease in APP/PS1 transgenic mice

**DOI:** 10.1038/s41598-020-71510-z

**Published:** 2020-09-03

**Authors:** Xueju Zhang, Weiwei Liu, Jie Zan, Chuanbin Wu, Wen Tan

**Affiliations:** 1 College of Pharmacy, Jinan University, Guangzhou, 510632 Guangdong China; 2 Postdoctoral Innovation Base, Zhuhai Yuanzhi Health Technology Co. Ltd, Hengqin New Area, Zhuhai, 519000 Guangdong China; 3 College of Biomedicine, Guangdong University of Technology, Higher Education Mega Center, Guangzhou, 510006 Guangdong China

**Keywords:** Biomarkers, Neurological disorders, High-throughput screening, Metabolomics

## Abstract

Alzheimer’s Disease (AD) is closely connected to aberrant lipid metabolism. However, how early AD-like pathology synchronously influences brain and plasma lipidome in AD mice remains unclear. The study of dynamic change of lipidome in early-stage AD mice could be of great interest for the discovery of lipid biomarkers for diagnosis and monitoring of early-stage AD. For the purpose, an untargeted lipidomic strategy was developed for the characterization of lipids (≤ 1,200 Da) perturbation occurring in plasma and brain in early-stage AD mice (2, 3 and 7 months) by ultra-high performance liquid chromatography coupled with quadrupole-time-of-flight mass spectrometry. Significant changes were detected in the levels of several lipid species including lysophospholipids, phosphatidylcholines (PCs), phosphatidylethanolamines (PEs) and Ceramides (Cers), as well as other related lipid compounds such as fatty acids (FAs), diacylglycerols (DGs) and triacylglycerols (TGs) in AD mice. In this sense, disorders of lipid metabolism appear to involve in multiple factors including overactivation of phospholipases and diacylglycerol lipases, decreased anabolism of lysophospholipids in plasma and PEs in plasma and brain, and imbalances in the levels of PCs, FAs and glycerides at different ages. We revealed the changing panels of potential lipid biomarkers with the development of early AD. The study raises the possibility of developing lipid biomarkers for diagnosis of early-stage AD.

## Introduction

AD is a progressive and chronic fatal neurodegenerative disease and the most common form of dementia, accounting for 60–80% of all dementia patients, and the number of patients with AD will reach 152 million until 2050^1,2^. Familial AD (an earlier onset < 65 years of age), is induced by mutations in the autosomal dominant genes encoding amyloid precursor protein (APP) and presenilins 1 and 2 (PS1 and PS2), which leads to the subsequent accumulation of β-amyloid (Aβ) in the brain^3–6^. The pathological accumulation and deposition of extracellular Aβ and aberrantly phosphorylated tau filaments in brain neurons finally lead to senile plaques and neurofibrillary tangles^5,7,8^. Double-transgenic mouse models co-expressing mutations in the human APP and PS1 genes are widely applied to explore underling pathophysiological mechanism of Aβ in early-stage AD. These mice generated Aβ plaques in the brain at 5–6 months old, although production of Aβ had been found as early as 3 months old^9^. APP/PS1 mice displayed progressive age-related memory impairments, which appeared as early as 7 months old^9–11^. In behavioristics testing, the mice showed significant deficiencies in measuring spatial memory and reference learning^10,11^. Thus, 2, 3 and 7-month-old APP/PS1 transgenic mice were selected to perform current study. Although APP/PS1 mice do not model all facets of human AD, they enable longitudinal investigations in laboratory but impossible in a clinical environment.

Lipidomics investigations of AD demonstrated that the pathology unbalanced lipid homeostasis and effected signal transduction^12,13^. Lipidome alteration reflects the situation of AD at certain time. Lipidomics technique has become a considerable potential as a discovery platform for identifying novel diagnostic lipid biomarkers for AD and other neurodegenerative diseases^14,15^. Lipidomics studies have revealed a series of biochemical disturbances in APP/PS1 mice^16–18^. Previous studies focused on untargeted and targeted metabolomics and lipidomics for AD^18–21^. Mass spectrometry has been considered as an important and powerful tool to conduct lipidomic studies for AD, including LC-QTOF/MS, LC-Orbitrap, LC–MS/MS, nano-ESI–MS, GC–MS and HPLC-ELSD^22–29^.

Although the abovementioned studies provided some valuable methodology, they suffered from limitations in many metabolomics investigations. For instance, a common limitation was a single time point, providing only the narrowest of windows through which to view and obtain reliable biological information^16,17^. On the other hand, the majority of previous studies were ill-considered to have the effect of early AD and gender on the lipidome^14,16^. In light of the deficiencies described above, the aim of our study was originally proposed to longitudinally study the dynamic change of lipid species in the APP/PS1 transgenic AD model over its lifespan from 2, 3 to 7 months old, and to monitor lipid disturbances close to the initial pathological impairment in brain of female AD mice as well as within the blood circulation, as depicted in Figure S1.

## Materials and methods

### Materials

HPLC-grade isopropanol and acetonitrile were bought from Merck (Darmstadt, Germany). LC–MS-grade ammonium formate and ammonium acetate were bought from Sigma-Aldrich (St Louis, USA). HPLC-grade ethyl acetate n-butanol and n-heptane were bought from Macklin (Shanghai, China). Water (Ω > 18.0) was purified by Milli-pore Milli-Q Ultra-pure water purification system (Alsace, France). All lipid standards in current study were bought from Avanti Polar Lipids lnc (Alabaster, AL, USA).

### Animal treatments

Specific-pathogen-free APP/PS1 mice, aged 2, 3 and 7 months and age-matched wild-type (WT) C57/BL6 mice were purchased from Animal Center of Guangdong Province (Foshan, Guangdong, China) and kept to acclimate for one week in a light and dark alternate, each cycle for 12 h with regulated temperature (23 ± 2 °C) and humidity (40–70%). APP/PS1 and WT mice were housed under the identical conditions and fed the same rodent maintenance diet. Animal studies were carried out in accordance with the Guiding Principles for Care and Use of Laboratory Animals, and the protocol was approved by the Ethics Committee of Experimental Animals of Jinan University (Certification number SCXK-2013-0002).

DNA of each mouse was extracted and determined according to the previously reported method^30,31^. Mice not expressing the transgene (APP/PS1) were used as WT controls. For this study, female AD model groups and age-matched WT controls (n = 8–13) were used.

Before the experiment, all mice were fasted for 12 h to avoid diet disturbance and fed with water. Mouse plasma samples were acquired by centrifuging blood, approximately 0.3 mL (4,000 rpm, 10 min, 4 °C) from the oculi chorioideae vein and stored at -80 °C. Mice were immediately sacrificed after withdrawing blood, and then mouse brain samples were instantly taken out from cranial cavity on ice, cleaned with normal saline, dehydrated on tissue surface, weighed and collected into individual new tubes and finally homogenized (50 Hz/s) in twofold volume of ice-cold normal saline for 1.0 min. The tissue homogenates were stored at –80 °C until further use.

### Sample preparation

Lipids were extracted by an optimized butanol-methanol method as described in the previous study to obtain the upper clear organic layer^32^. Briefly, each sample (20 μL) (including plasma and brain homogenate samples) was successively treated with 200 μL of solution A (3:1, butanol-to-methanol volume ratio) and solution B (3:1, n-heptane-to-ethyl acetate volume ratio), and next layered by adding 200 μL of ammonium acetate (50 mM) and further handled by ultrasound (60 Hz, 200 W) for 10 min. The upper organic layer was obtained by centrifugation (6,000 rmp) for 10 min 4 °C and transferred to a new tube and dried under vacuum. Finally, all dried samples were reconstituted with acetonitrile/ isopropanol/water (100 μL, 3:4:1, v/v/v), treated by ultrasound (60 Hz, 200 W) for 5 min and the clear solutions were removed for LC–MS analysis. Equal aliquots of each sample were pooled and mixed to make quality control (QC) samples for further analysis.

### UHPLC-QTOF/MS analysis

A Thermo Scientific Dionex UltiMate 3,000-UHPLC system (Thermo Fisher scientific, CA, USA) coupled with a QTOF/MS spectrometer with an electrospray ion source (ESI) (Bruker Daltonics Inc, Billerica, MA) was utilized in present study. UHPLC-QTOF/MS analysis was performed based on our previous investigation^32^. The chromatographic separation was achieved on an ACQUITY UPLC CSH C18 column (2.1 mm × 100 mm, 1.7 μm) from Waters Technologies (Milford, MD, USA). The column temperature was set at 55 °C. A binary mobile phase system consisted of (A) acetonitrile containing 10 mM ammonium acetate and (B) acetonitrile/isopropanol (10:90, v/v) containing 10 mM ammonium acetate. Mobile Phase constitution was changed as followed: initiation at 40% B followed by a linear gradient to 43% B over 2 min, turned to 45% B at 2.1 min, turned to 48% B at 12 min, reached 60% B at 12.1 min, rose to 100% B at 18 min, returned to 40% B at 18.1 min and lasted 1.9 min. The flow rate was set at 0.4 mL·min^−1^. The injection volume was 20 μL. The analysis of mass spectrometry was operated using an electrospray ionization source (ESI) in the negative and positive mode. The optimized parameters were set under the negative mode as follows: capillary voltage, 3.5 kV; charging voltage, 2.0 kV; end plate offset, 500 V; dry temperature, 250 °C; nebulizer pressure, 2.0 bar; dry gas, 6 L·min^−1^, funnel 1 RF 350 Vpp; funn el 2 RF 600 Vpp; multipole RF 700 Vpp; collision RF 1,200 Vpp; transfer time, 80 μs and deflection delta 70 V; Under the positive ion mode, capillary voltage, 4.5 kV; charging voltage, 2.0 kV; end plate offset, 500 V; dry temperature, 250 °C; nebulizer pressure, 2.0 bar; dry gas, 7 L·min^−1^, funnel 1 RF 350 Vpp; funnel 2 RF 600 Vpp; multipole RF 1,200 Vpp; collision RF 1,000 Vpp; transfer time, 70 μs and deflection delta 70 V. Nitrogen was used as the cone and desolvation gas, and argon was used as the collision gas. Collision energies were set at 7 eV for low energy under negative and positive modes and linear energy change from 25 to 75 eV with molecule weight changing for high energy, respectively under negative and positive modes for Auto-MS/MS mode (automatically selecting the first 10 parent ions with the strongest intensity ((*m/z*, 50–1,200 Da; absolute intensity, greater than 5,000 cts ; width, ± 0.5) in the respective time slice to acquire their fragment ions(acquisition control including spectra rate, 1.0 Hz; dynamic ms/ms spectra acquisition: target intensity, 1,000 cts; maximal rate, 45 Hz and minimal rate, 9 Hz). Accurate mass was maintained by introduction of a micro-syringe bump containing sodium formate at a concentration of 10 mM in 50% aqueous isopropanol and a rate of 20 μl·min^−1^ before the chromatographic separation. Data was acquired and processed by otofControl 5.0 and Data analysis 5.0 software, respectively.

### Data processing and statistical analysis

Data processing and statistical analysis were performed according to the previous reference^32^. Briefly, the cluster peaks (theoretical value, *m/z*) of sodium formate introduced before the chromatographic separation were used to recalibrate the *m/z* of all peaks in raw data. Next, the corrected peak areas were utilized for further analysis. Progensis QI v2.0 (Nonlinear Dynamics, Newcastle, U.K.) and EZinfo 3.0.3 (Waters, Milford, USA) were employed to visualize, process, and interpret multi-dimension LC–MS data. The corrected LC–MS data were imported to Progensis QI for peak picking and alignment. Data was further normalized using total ion intensity. Analysis was explored by using multivariate statistical methods, including principal components analysis (PCA) and orthogonal partial least square and partial least square-discriminant analysis (OPLS/PLS-DA) and further confirmed using analysis of variance (ANOVA) in EZinfo. The peak height intensity of the differential lipid metabolites was compared by *t*-test using statistical software to confirm the biomarker alterations between WT and AD groups, and only the intensity with P value threshold set at 0.05, fold change at 1.3, and variable coefficient (CV) at 30 could be considered as the potential biomarkers. Difference between AD and WT groups is considered statistically significant when P value is less than 0.05.

For visualization of pathway analysis, Metaboanalyst (Metabolic Pathway Analysis, https://www.metaboanalyst.ca/faces/ModuleView.xhtml) based on database source including KEGG and HMDB, was used to help relevant pathway analysis and visualization of potential biomarkers with its topological characteristics in this work.

### Metabolite identification

Potential lipid biomarkers were identified through matching the experimental retention time (± 0.1 min), accurate mass and tandem mass spectra (MS/MS), combing with those available in metabolomic databases (HMDB and LIPIDMAPS) with a mass accuracy of ± 5 ppm for parent ions and ± 20 ppm for fragment ions, and, then confirmed with marketable standard compounds when available. Different classes of lipids were confirmed based on characteristic fragmentation patterns previously described, including PCs and lysophosphatidylcholines (lysoPCs) (*m/z* 184.07, 104.10 and 86.10 for ESI + and *m/z* 168.04 for ESI−) , PEs and lysophosphatidylethanolamines (lysoPEs) (*m/z* 196.04 for ESI + and 140.01, 196.07, 283.40 for ESI−) or phosphoserine moiety and choline (*m/z* 168.04 for ESI−), ethanolamine (*m/z* 196.07 for ESI-) and serine derived lipids^33–35^. Furthermore, fragmentation of glycerides (di- and triacylglycerol) occurs through the release of fatty acids generating different types of ions, which show characteristic *m/z* values according to the fatty acid attached to the glycerol backbone^36^. On the other hand, cholesteryl esters can be easily identified by means of an abundant fragment ion at *m/z* 369.40 induced upon collision induced dissociation^37^. Free fatty acids were also confirmed with characteristic fragments described in the literature^38–40^.

## Results and discussion

### Metabolic profiles of plasma and brain lipidome of early-stage AD mice

An initial principal component analysis (PCA) plot was generated with data from plasma and brain using the different MS techniques (UHPLC-ESI(−/ +)/Qtof/MS) coupled with chromatographic separation (Figure S2 in supplementary material) in order to check trends and outliers to ensure grouping of all samples. These models generated reliable values for the quality parameters R2 and Q2, with a variance explained close to 100% and variance predicted above 80% for all models.

**Figure 1:**
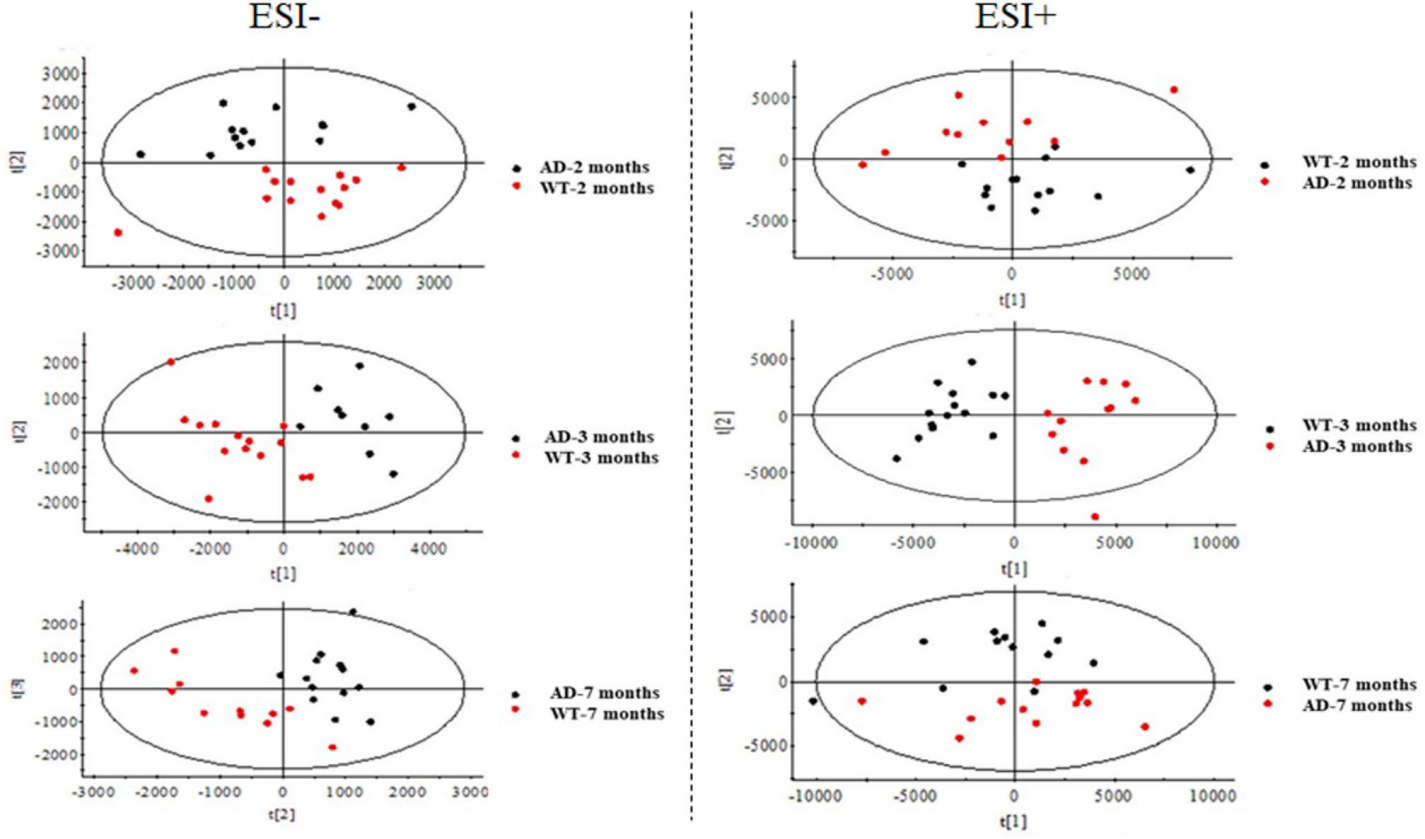
PCA plots of statistical model for lipidomic data from plasma samples in the negative and positive ion modes of UHPLC-QTOF/MS.

**Figure 2:**
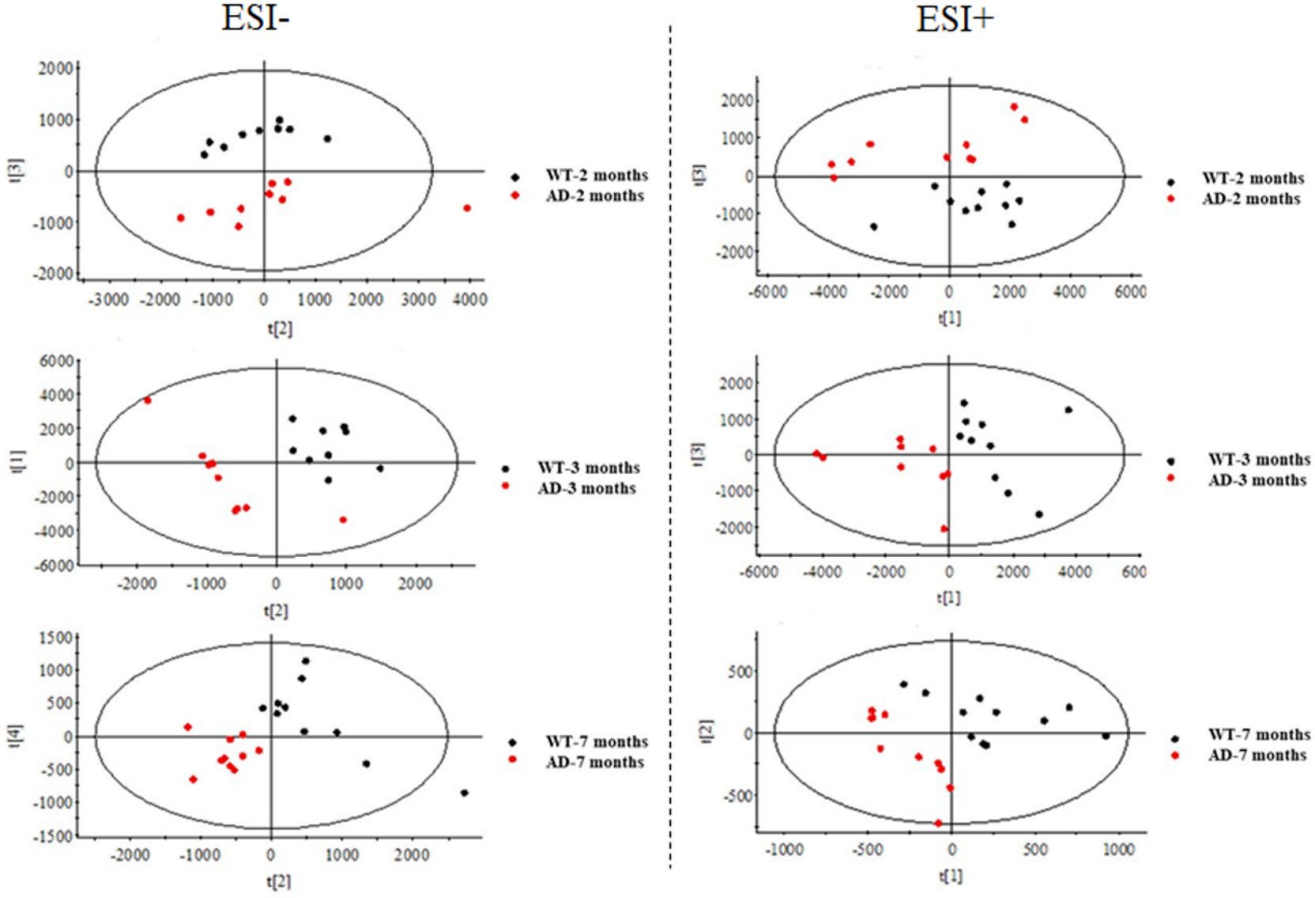
PCA plots of statistical model for lipidomic data from brain samples in the negative and positive ion modes of UHPLC-QTOF/MS.

These assessments were made to ascertain the overall extent of lipidome changes in brain and plasma at each age point for all samples. Multivariate analysis was used to build models to discriminate all groups (WT and AD groups for 2, 3 and 7 months) analyzed for plasma (Fig. 1) and brain (Fig. 2). The score plots showed that it was possible to distinctly discern WT and APP/PS1 mice at all ages. However, it was more difficult to distinguish WT from APP/PS1 (7 months) as there was some degree of overlap in the plasma samples in the positive mode. In the three-dimensional (3D) score plots, we found that there were changing trends among different groups (Figure S3 in supplementary material). The results of 2D and 3D score plots of PCA in this study showed possible effects of early-stage AD on lipidome in mouse blood and brain.

### Potential lipid biomarker panel of AD progression

**Figure 3:**
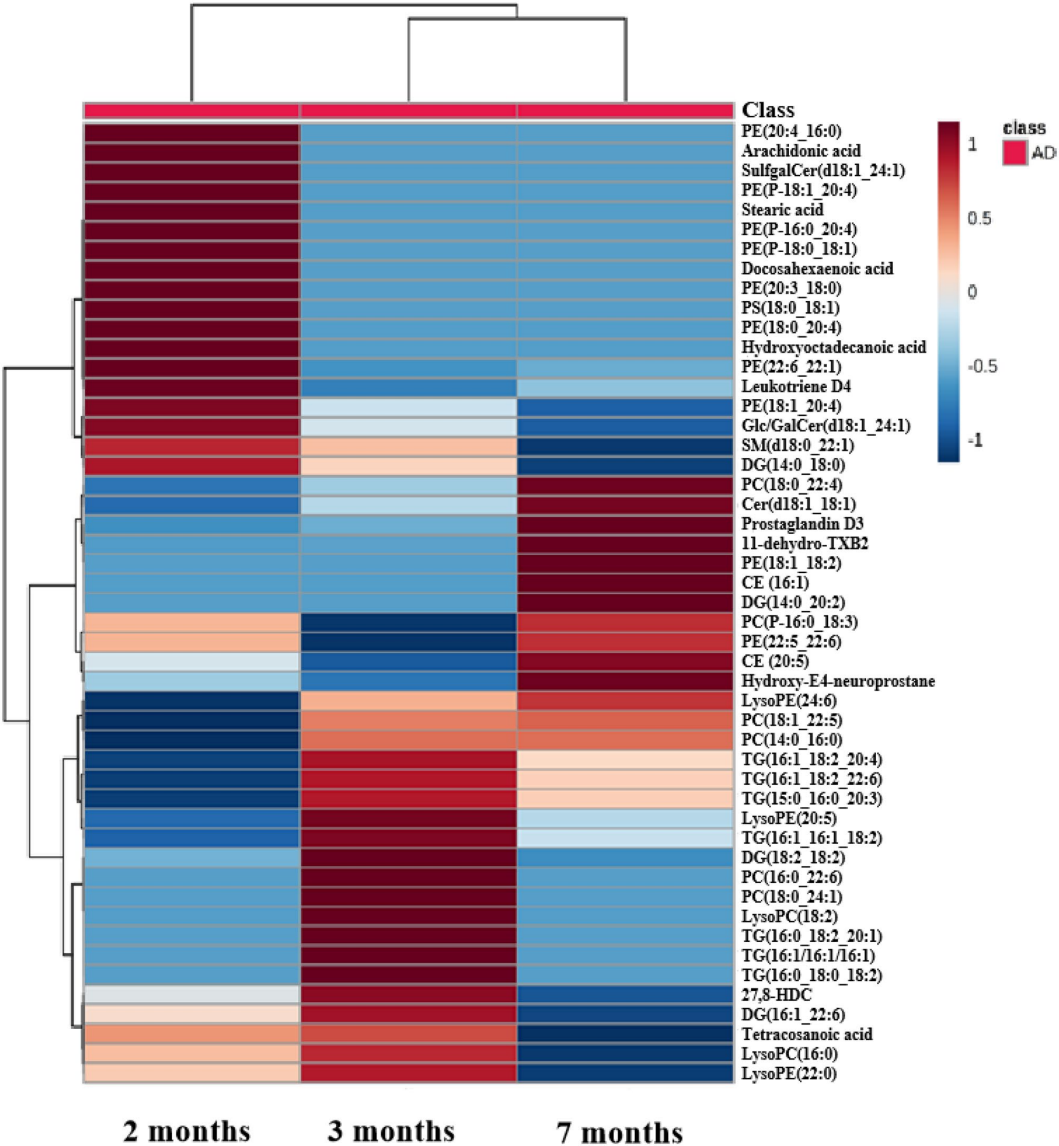
Heatmap clustering was visualized for fold change of all potential lipid biomarkers in plasma and brain of AD mice, aged with 2, 3, and 7 months, showing the level of relative increase (brown) and decrease (blue).

In lipid extraction, recoveries of lipid standards were checked to confirm the efficiency of lipid extraction for latter unbiased lipid analysis and results showed that recoveries of different lipid species were more than 50% (Table S1 in supplementary material). Potential lipid biomarkers in brain and plasma of early-stage AD mice at the ages of 2, 3 and 7 months were further extracted, based on multivariate statistical analysis according to the conditions that the parameters of altered lipids satisfied fold change ≥ 1.3 or ≤ 0.77 (intensity ratio, AD/WT), CV (Coefficient of Variation) ≤ 30%, ANOVA (*p*) < 0.05, VIP ≥ 1.0 at any age. The detailed results were presented in Tables S2-1, S2-2, S2-3 and Table S3 in supplementary material. Meanwhile, a heatmap (Fig. 3) and bar plots (Figure S4 and S5) were presented based on fold change of all potential biomarkers identified lipid compounds. As depicted in Fig. 3, the clustering provided an overview of all potential lipid biomarkers in plasma and brain at 2, 3 and 7-month-old APP/PS1 mice, showing the fluctuant levels of relative increase (brown) and decrease (blue).

These lipid biomarkers in brain and plasma of early-AD mice provided the opportunity to elucidate possible biochemical pathways affected in the APP/PS1 mice, thus allowing a better understanding about the disease pathology. Numerous lipid compounds were significantly disturbed in plasma and brain from APP/PS1 mice. Lysophospholipids are generated by the action of phospholipase A_1_ (PLA_1_) and phospholipase A_2_ (PLA_2_) and are either hydrolyzed by lysophospholipase or used to regenerate phospholipids in the remodeling pathway^41^. The most notable finding was an abnormal metabolism of phospholipids regarding changed levels of lysoPCs, lysoPEs and PCs, as well as other related compounds such as DGs, TGs, cholesteryl esters (CEs) and other catabolites in plasma and brain of APP/PS1 mice in current study. Abnormal metabolism of membrane phospholipids caused by over-activated phospolipases activity, mainly PLA_2_ , has been traditionally considered as a key pathological hallmark in the development of Alzheimer’s disease^34^. Recent studies performed in serum samples from human AD patients and mice also pointed to the implication of an altered fatty acid composition of phospholipids containing decreasing polyunsaturated fatty acids (PUFA) and increasing saturated species^33,42,43^. During the remodeling of phospholipids, lysophospholipids are transiently generated by the action of PLA_2_, but they are rapidly acylated with acyl-CoA in the diacylation reacylation cycle for the maintenance of the normal and essential neural membrane composition^41^. However, it is discovered that lysophospholipids are not only intermediates in metabolism of glycerides, but they serve as mediators in multiple neuronal pathways involved in neurobiology of AD ^42^. In this sense, we detected overall decrease of different lysophospholipids such as LysoPC(18:2), LysoPC(16:0) and LysoPE(24:6), LysoPE(22:0) , LysoPE(20:5) in plasma from AD mice at different ages (2, 3 and 7 months) in accordance with previous investigations in serum of transgenic mice of AD and human AD^33,42,44^. Furthermore, substantial decrease of different PEs (including PE(20:3_18:0), PE(22:6_22:1), PE(22:6_22:0), PE(P-16:0_20:4), PE(P-18:1_20:4), PE(P-18:0_18:1)) in plasma and brain of the APP/PS1 model (2, 3 and 7 months) in this study were observed, which had the similar situation with previous studies in different brain region of AD mouse^34,45,46^. The reason of such decrease in phospholipids may be the stimulation of various isoforms of PLA_2_ including PE-PLA2, cytosolic PLA_2_(cPLA_2_) and secretory PLA_2_(sPLA_2_), which leads to the increased activities of PLA2 isoforms in AD brain^47^. The decrease of PCs (including PC(P-16:0_18:3), PC(14:0_16:0) and PC(18:0_24:1)) in plasma and brain of the APP/PS1 mice with the ages of 2, 3 and 7 months were consistent with the decrease in brain of 6-month-old AD transgenic mice in these published literatures^48,49^. At the same time, there were some increased PCs (including PC(16:0_22:6), PC(18:1_22:5) and PC(18:0_22:4)) in AD. The increase of PEs (including PE(20:4_16:0), PE(18:1_18:2), PE(18:1_20:4), PE(22:5_22:6) and PE(15:0_22:4)) and PCs denotes a profound membrane remodeling in the APP/PS1 mice^34^.

Apart from the glycerolipids and glycerophospholipids, we also found that SM(d18:0_22:1) belonging to sphingolipids, was significantly altered. Sphingolipids are not only structural components of biological membranes, but also involve in many important physiological and pathophysiological processes including cellular growth, cell adhesion, differentiation, migration and apoptosis^50^. There were some mutative ceramides and its analogues including ceramide(d18:1_18:1) (Cer (d18:1_18:1)), 3-O-Sulfogalactosylceramide(d18:1_24:1) (SulgalCer(d18:1_24:1)) in brain, glucosyl- or galactosyl-ceramide(d18:1_24:1)(Glc/GalCer(d18:1_24:1)) in plasma. It is interesting that the level of Cer (d18:1_18:1) distinctly decreased at 2 and 3 months old and then increased in the brain of AD mice at 7 months old but the previous study reported the level of Cer(d18:1_18:1) significantly increased in blood serum of AD subjects^48^, suggesting such reverse change at early-stage AD. These results implied that ceramides might be glycosylated and sulfated to form Glc/GalCer(d18:1_24:1) and SulgalCer(d18:1_24:1), respectively and the enzymatic activity involving in these metabolism processes could be changed in APP/PS1 mice. In our study, the significantly decreased Glc/GalCer(d18:1_24:1) indicated that the synthesis of Glc/GalCer (d18:1_24:1) by glucosylceramide/galactosylceramide synthase might be weakened, which was opposite to the case of SulgalCer(d18:1_24:1). These results implied the perturbed sphingolipid metabolism in APP/PS1 mice and revealed these dysregulated lipid metabolic pathways in a more targeted and specific way, which perhaps provide more clues for early-AD pathology.

Diacylglycerol lipases have been demonstrated that they have higher (six to eight times) enzymic activity in plasma membrane and synaptosomal plasma membrane from different brain regions of Alzheimer’s patients than normal subjects^12^. Furthermore, some evidences demonstrated a role of abnormal phospholipase C (PLC) and D (PLD) in processes associated with degradation of phospholipids in AD^51,52^. These enzymes hydrolyze phosphoester bonds in the hydrophilic group of phospholipids, thus releasing diacylglycerols. DGs could be further hydrolyzed into monoacylglycerols by monoacylglycerol lipase such as in the process of degradation of PC^12^. In this context, the elevation of total levels of diacylglycerols in serum from AD patients could be considered as potential marker of over-activated PL C/D^53,54^. In our study, the increased DGs (including DG(16:1_22:6), DG(18:2_18:2), DG(14:0_20:2)) were observed in the plasma and brain of AD mice (2, 3 and 7-month old) to support the above-mentioned biological hypothesis.

Moreover, the abovementioned changes and abnormalities in lipid metabolism were also reflected in other compounds not directly related to membrane destabilization processes, including free FA, CE and TG. The accumulation of TG suggests a serious hyperlipidemia, one of the most important vascular risk factors that have been associated with the development of AD. Furthermore, several investigations have proved relationships between Alzheimer’s disease and high levels of lipids, principally triglycerides in both mice and human blood^43,55^. In this study, all of TGs (including TG(16:1_18:2_22:6), TG(16:1_16:1_16:1), TG(16:1_16:1_18:2), TG(16:1_18:2_20: 4), TG(15:0_16:0_20:3), TG(16:0_18:0_18:2) and TG(16:0_18:2_20:1)) were observed to increase in plasma of AD mice, indicating the accumulation of TGs in blood circulation.

In addition, free fatty acid (including docosahexaenoic acid, arachidonic acid, hydroxyoctadecanoic acid, stearic acid and tetracosanoic acid), cholesteryl ester(CE(20:5), CE (16:1)), cholesterol(27alpha-hydroxy-8-dehydrocholesterol(27,8-HDC)), prostaglandin D3, leukotriene D4, hydroxy-E4-neuroprostane were also found to have abnormal change in plasma and brain of AD mice in this study. In these metabolites, leukotriene D4, prostaglandin D3 and hydroxy-E4-neuroprostane in AD brain involved in the process of inflammation cascade^56–58^, indicating inflammation might occur in the brain of AD mice. Decreased free FAs including docosahexaenoic acid, arachidonic acid, hydroxyoctadecanoic acid, stearic acid and tetracosanoic acid were detected in the plasma and brain of AD mice in this investigation. Previous studies also accordingly demonstrated that several free fatty acids decreased in serum of APP/PS1 mice, plasma and brain of AD patients^33,59–62^. The levels of bile acids are clearly disturbed during the development of AD pathology^63^. In our study, 27,8-HDC significantly increased in plasma of 2- and 3-month-old AD mice.

Also, we found that early AD pathology significantly altered lipid constitutes in mouse plasma as well as lipid metabolism in the brain. It is interesting that 11-dehydro-thromboxane B2(11-dehydro-TXB2), prostaglandin D3 and Cer(d18:1_18:1) decreased in AD brain at 2 and 3 months, and increased at 7 months. It is notable that hydroxy-E4-neuroprostane were observed that its level decreased in AD brain at 2 months but increased at 7 months. However, the changed level for leukotriene D4 was just opposite in the brain of early-stage AD mice. The reason of such contrary alterations is unclear and needs to further be confirmed. It is speculated that the pathology of early-stage AD affects the metabolism of multiple pathways containing these abnormal lipid metabolites.

Taken together, much more potential lipid biomarkers in plasma than in brain of AD mice were observed to reflect the pathological state of the early-stage AD. PEs (∼20% of total phospholipids) and PCs (∼45%) are major components of the cell membrane in the brain and play essential roles involved in neural membrane formation, trans-bilayer movement, intraneuronal signal transduction, recognition and engulfment of cells, maintaining normal mitochondrial morphology^64–67^. The levels of PC(14:0_16:0) (2,3 and 7 month old) and PC(18:0_24:1) (3 month old) decreased in the brain of AD mice in this study, indicating that they undergo flux. However, levels of PC(14:0_16:0) and PC(18:0_24:1) were not found to increase in the plasma. Meanwhile, PE(22:5_22:6) (2 and 7 month old) and PE (15:0_22:4) (7 month old) in the brain undergo influx due to their increased levels. PE(20:4_16:0) (2 month old), PE(18:1_20:4) (2, 3 and 7 month old), PE(18:1_18:2) (7 month old) and PE (22:6_22:1) (2, 3 and 7 month old) in the plasma also increased in AD mice but they had no significant fluctuation in the brain. It is speculated that these significantly altered PCs and PEs in the brain could be diluted in the blood circulation and show no significant difference in the plasma. Such differences of potential biomarkers between brain and plasma of AD mice at different month ages, as discussed previously, could provide an opportunity to view the pathological progression of the early-stage AD.

We also found that some disturbances were frequently transient and others were persistent with AD progression at 2, 3 and 7 months, as described above in this study. Therefore, not all potential biomarkers can be selected to serve as decent biomarkers to monitor and diagnose the progression of early AD. Many potential lipid biomarkers in AD mice could not be significantly changed at all early stages (2, 3 and 7 months) possible because the pathophysiology of early-stage AD slightly and finitely disturbs the lipid metabolism. However, a biomarker panel could be selected to reflect overall situation of the pathophysiology of AD at certain timepoint^68^. These were just evident from our broader timeframe of longitudinal assessment compared with other studies^34,45^.

### Metabolic pathway and flux analysis of potential biomarkers

**Figure 4:**
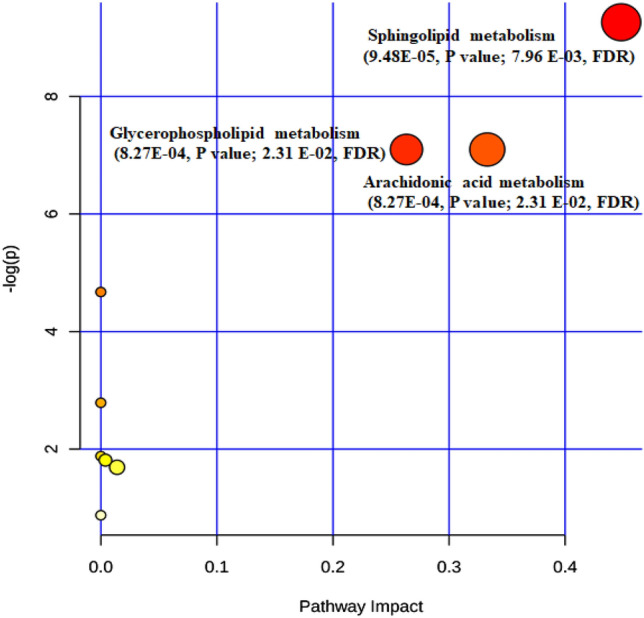
Pathway analysis overview, where each node represents an altered metabolic pathway in APP/PS1 mice and its size indicates the impact of this pathway. FDR notes False Discovery Rate.

Metabolic pathway analysis (MPA) and multiple comparison testing by the identified biomarker candidates demonstrated that there were significant changes (*p* < 0.05) in lipid metabolism including glycerophospholipid, sphingolipid, arachidonic acid metabolism and biosynthesis of unsaturated fatty acid in AD mice. As presented in Fig. 4, sphingolipid metabolism (0.45, impact; 9.48E-05, *p* value; 7.96 E−03, FDR), glycerophospholipid metabolism (0.26, impact; 8.27E-04, *p* value; 2.31 E−02, FDR) and arachidonic acid metabolism (0.33 impact; 8.27E-04, P value; 2.31 E-02, FDR) were significantly impacted in early AD mice model. These results were in accordance with the altered metabolic pathway in AD mice and patients^31,40,48^. Arachidonic acid metabolism containing altered arachidonic acid, phosphatidylcholine and leukotriene D4 had significant difference from WT mice, indicating that inflammation might occur in AD mice brain, as known that inflammatory cascade might induce AD as a kind of biological hypothesis^69^.

**Figure 5:**
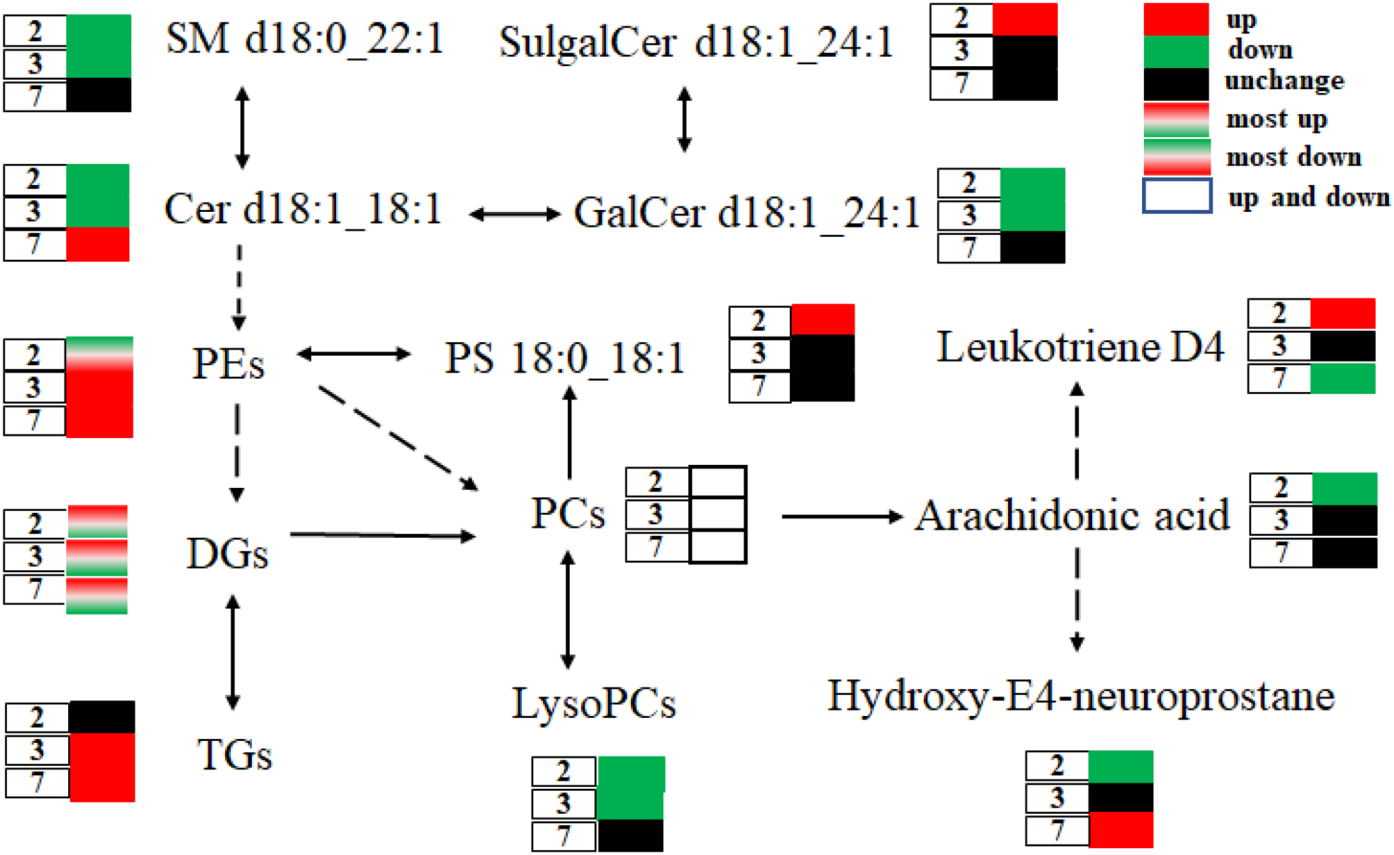
Metabolic flux of abnormal lipid metabolites in the early-stage AD mice with 2, 3 and 7 months old.

In the analysis of metabolic flux of potential lipid biomarkers, the metabolic flux were constructed to show the metabolic relationships of these changed metabolites including lysoPC, PE, PC, DG, TG and other lipid compounds, including PS(18:0_18:1), Cer(d18:1_18:1), Glc/GalCer(d18:1_24:1), SulgalCer(d18:1_24:1), arachidonic acid, Leukotriene D4 and hydroxy-E4-neuroprostane in APP/PS1 mice at the ages of 2, 3 and 7 months, as depicted in Fig. 5. It is notable that Glc/GalCer(d18:1_24:1) in plasma and SulgalCer(d18:1_24:1) in brain are found to be altered after glycosylation and sulfation of ceramide in the current study, indicating that pathology of early-stage AD may affect related enzymes of glycosylation and sulfation metabolism of ceramides. Such compounds might become new biomarkers for early diagnosis of AD due to the significant change occurring at 2 and 3 months old.

## Conclusion

This study originally provides a systematic approach to investigate pathophysiology of early AD to synergistically disclose dynamic change of potential lipid biomarker panels in mouse plasma and brain, and also provides strong evidence to lipid metabolic alterations in early-stage AD mice. The study enhances the possibility of developing lipid biomarkers for diagnosis and monitoring of early-stage AD.

## Supplementary information

Supplementary Information.
