# Author Correction: Untargeted lipidomics reveals progression of early Alzheimer’s disease in APP/PS1 transgenic mice

**DOI:** 10.1038/s41598-021-96907-2

**Published:** 2021-08-26

**Authors:** Xueju Zhang, Weiwei Liu, Jie Zan, Chuanbin Wu, Wen Tan

**Affiliations:** 1 College of Pharmacy, Jinan University, Guangzhou, 510632 Guangdong China; 2 Postdoctoral Innovation Base, Zhuhai Yuanzhi Health Technology Co. Ltd, Hengqin New Area, Zhuhai, 519000 Guangdong China; 3 College of Biomedicine, Guangdong University of Technology, Higher Education Mega Center, Guangzhou, 510006 Guangdong China

Correction to: *Scientific Reports* 10.1038/s41598-020-71510-z, published online 03 September 2020

The Author Contributions section in the original version of this Article was incomplete.

“Dr. Xueju Zhang designed the study, developed the method, wrote the first draft, revised the draft, commented on it and finalized it. Mr. Weiwei Liu collected the samples, prepared the samples and run the methods. Dr. Jie Zan analyzed the data. Dr. Wen Tan and Dr. Chuanbin Wu supervised the study. Dr. Jie Zan, Dr. Wen Tan and Dr. Xueju Zhang provided the funding supports for the study.”

now reads:

“Professor Wen Tan participated in designing the study and provided experiment support. Dr. Xueju Zhang developed the method, wrote the first draft, revised the draft, commented on it and finalized it. Mr. Weiwei Liu and Dr. Xueju Zhang collected the samples, prepared the samples and run the methods. Dr. Jie Zan and Dr. Xueju Zhang analyzed the data. Professor Wen Tan and Professor Chuanbin Wu supervised the study. Professor Wen Tan, Dr. Jie Zan and Dr. Xueju Zhang provided the funding supports for the study.”

The original Article has been corrected.

