# Supplementary material for: Untargeted lipidomics reveals progression of early Alzheimer’s disease in APP/PS1 transgenic mice
Source: Sci Rep. 2020 Sep 3;10:14509. doi: 10.1038/s41598-020-71510-z (PMC7471266; doi:10.1038/s41598-020-71510-z)
Supplement: Supplementary file 1 — Supplementary Information. [file 41598_2020_71510_MOESM1_ESM.docx]

Untargeted Lipidomics Reveals Progression of Early Alzheimer’s Disease in APP/PS1 Transgenic Mice

Xueju Zhang^1,2,∗^, Weiwei Liu^3^, Jie Zan^3^, Chuanbin, Wu^1^, Wen Tan^3,∗^

^1^College of Pharmacy, Jinan University, Guangzhou, Guangdong 510632, China.

^2^Postdoctoral Innovation Base, Zhuhai Yuanzhi Health Technology Co. Ltd, Hengqin New Area, Zhuhai, Guangdong 519000, China.

^3^College of Biomedicine, Guangdong University of Technology, Higher Education Mega Center, Guangzhou, Guangdong 510006, China.

∗To whom Correspondence should be addressed:

E-mail address: [xjzhangjack@126.com](mailto:xjzhangjack@126.com)(X.J. Zhang), Tel: +86-20-39325249; [went@gdut.edu.cn](mailto:went@gdut.edu.cn)(W. Tan), Tel: +86-20-39380669


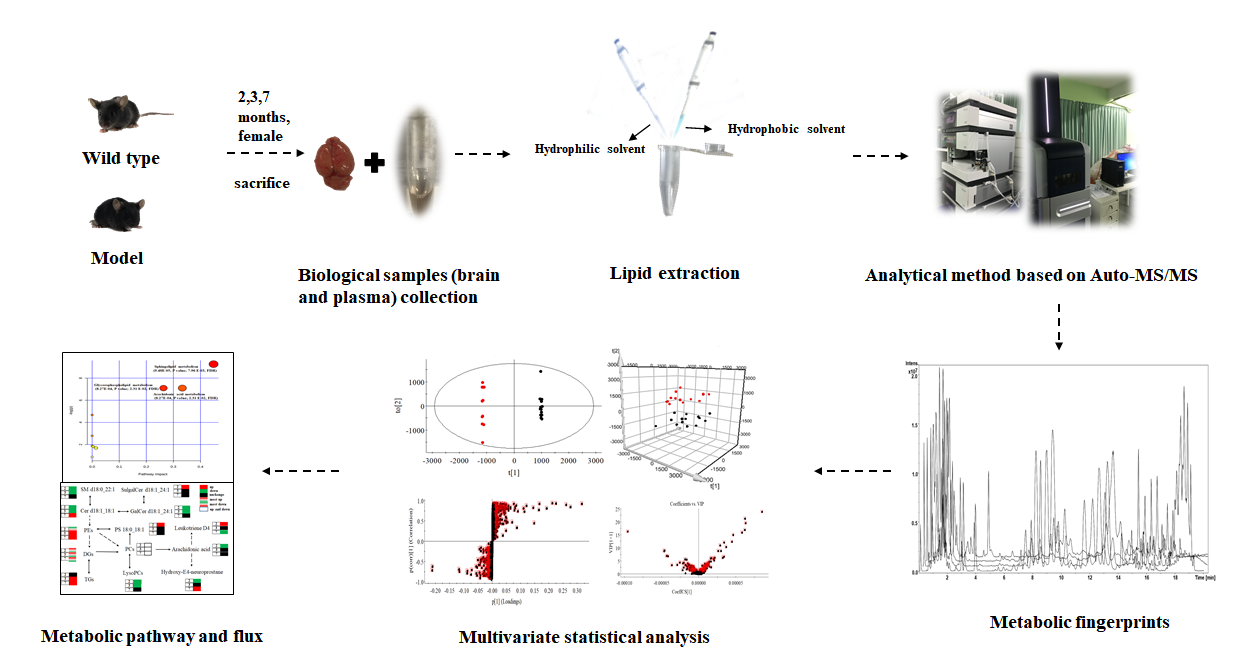


**Figure S1.** A strategy for longitudinally study of the profile of dynamic change of lipid species in the APP/PS1 transgenic AD model over its lifespan from 2, 3 to 7 months old.

**
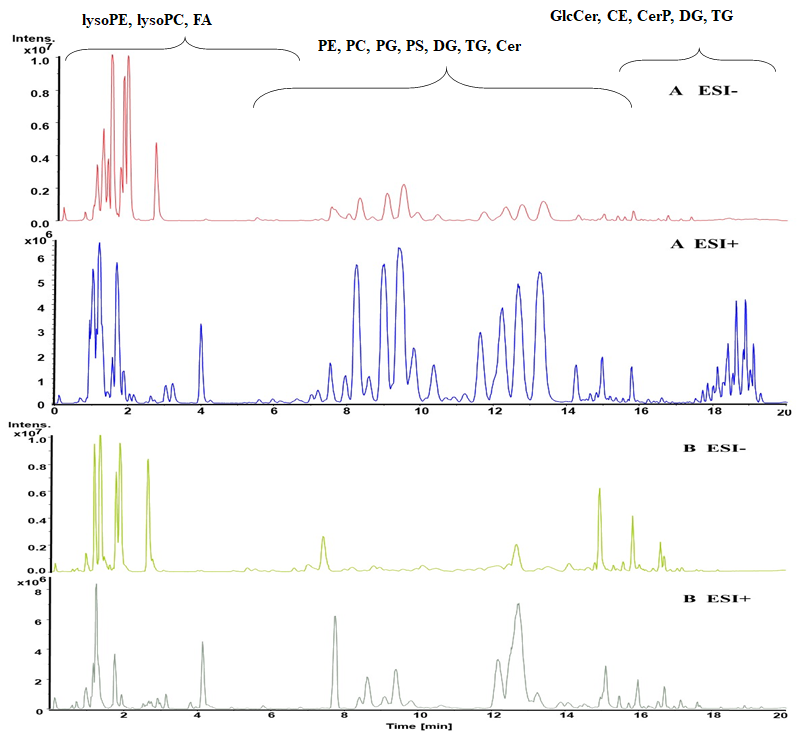
**

**Figure S2.** Chromatograms of lipidome from mouse plasma (A, ESI-, ESI+) and brain (B, ESI+, ESI-) in the both positive and negative modes.


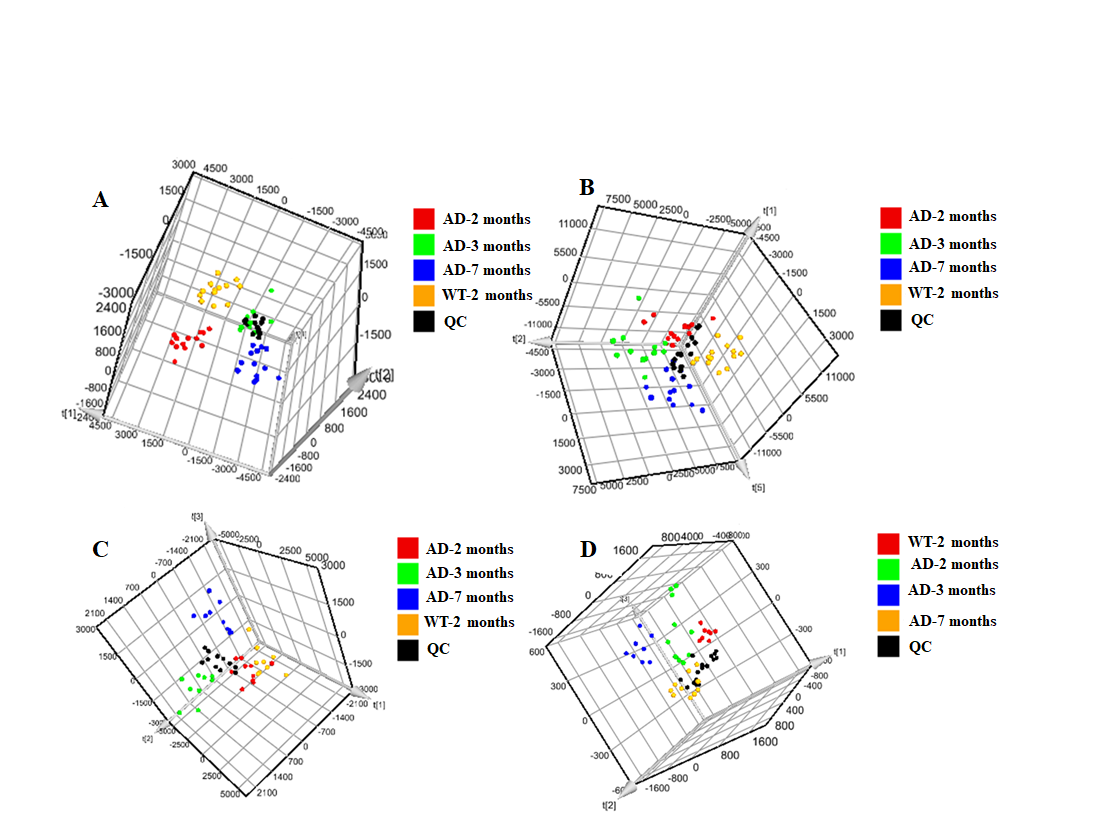


**Figure S3.** Three-dimensional score plots by PLS-DA of the MS data of feature ions, derived from plasma (**A**, ESI- and **B**, ESI+) and brain (**C**, ESI+ and **D**, ESI-) of AD mice at 2, 3 and 7 months old and WT mice at 2 months old.

**
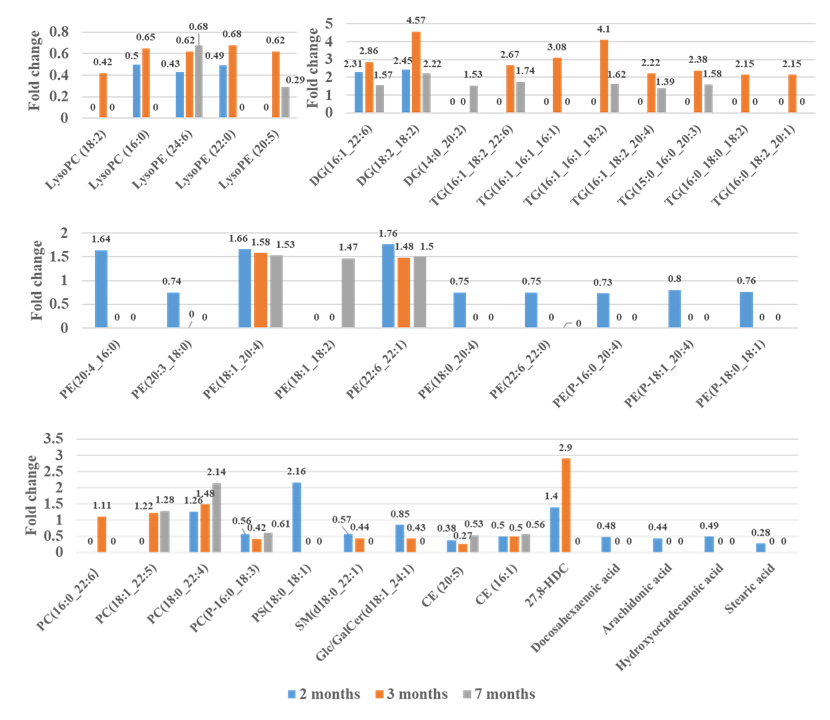
Figure S4.** Bar plot of potential lipid biomarkers in the plasma of early-stage AD mice (2, 3 and 7 months).


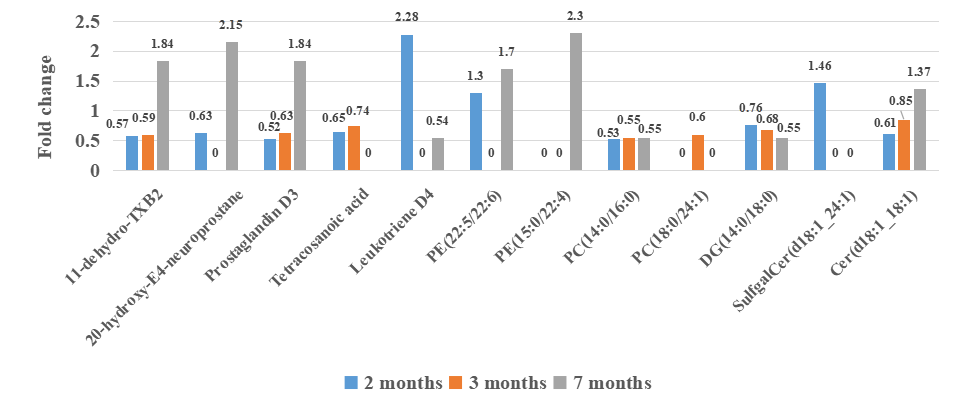


**Figure S5.** Bar plot of potential lipid biomarkers in the brain of early-stage AD mice (2, 3 and 7 months).

| Lipid name | Recovery (%, mean±SEM, n=3) | |
| --- | --- | --- |
|  | In plasma matrix | In brain matrix^1^ |
| PE(18:0/18:0) | 58.83±7.20 | 59.22±8.91 |
| PC(17:0/17:0) | 73.11±7.18 | 77.76±12.32 |
| PG(14:0/14:0) | 66.71±1.64 | 74.10±1.41 |
| PI(16:0/18:1) | 65.45±4.75 | 52.42±7.06 |
| PS(14:0/14:0) | 67.03±6.26 | 70.41±0.17 |
| Cer(d18:1/17:0) | 73.21±2.50 | 67.85±14.05 |

**Table S1.** Recovery of different lipid species in plasma and brain matrix.

| Potential biomarker  (possible identification） | Adduct ion | MS/MS | Retention time (min) | m/z  (mass error, ppm) | Age (month) | | | | | |
| --- | --- | --- | --- | --- | --- | --- | --- | --- | --- | --- |
|  |  |  |  |  | 2 | | 3 | | 7 | |
|  |  |  |  |  | Fold change | P value | Fold change | P value | Fold change | P value |
| LysoPC(18:2) | [M+Formate]^-^ | 123.0015,  279.2609 | 2.71 | 564.3304  (-0.61) | N.S. | >0.05 | 0.42 | 5.05E-03 | N.S. | >0.05 |
| LysoPC(16:0) | [M+Acetate]^-^ | 123.0036,  255.2406 | 4.96 | 554.3461  (-0.43) | 0.50 | 7.35E-05 | 0.65 | 2.41E-03 | N.S. | >0.05 |
| LysoPE(24:6) | [M-H]^-^ | 140.0931,  331.3207,  337.3150 | 3.92 | 552.3106  (1.79) | 0.43 | 2.73E-05 | 0.62 | 4.25E-04 | 0.68 | 1.93E-02 |
| LysoPE(22:0) | [M+Formate]^-^ | 295.3012,  321.3271,  339.3301 | 6.30 | 582.3778  (0.29) | 0.49 | 2.89E-05 | 0.68 | 1.35E-02 | N.S. | >0.05 |
| LysoPE(20:5) | [M+Formate]^-^ | 283.3021,  301.3047 | 6.90 | 544.2675  (-1.22) | N.S. | >0.05 | 0.62 | 8.31E-04 | 0.29 | 4.43E-06 |
| PE(20:4_16:0) | [M-H]^-^ | 255.3304,  303.3215 | 9.68 | 738.5047  (-4.30) | 1.64 | 1.63E-05 | N.S. | >0.05 | N.S. | >0.05 |
| PE(20:3_18:0) | [M-H]^-^ | 283.2701,  305.2616,  502.3015 | 9.87 | 768.5517  (-4.19) | 0.74 | 2.52E-05 | N.S. | >0.05 | N.S. | >0.05 |
| PE(18:1_20:4) | [M-H]^-^ | 281.2701,  303.2518 | 10.08 | 764.5204  (-4.16) | 1.66 | 5.29E-05 | 1.58 | 1.11E-04 | 1.53 | 1.21E-02 |
| PE(18:1_18:2) | [M-H]^-^ | 279.2301,  281.2217 | 10.58 | 740.5256  (-3.40) | N.S. | >0.05 | N.S. | >0.05 | 1.47 | 1.96E-02 |
| PE(22:6_22:1) | [M-H]^-^ | 327.2602  337.3109 | 13.34 | 844.5836  (-3.03) | 0.82 | 1.07E-05 | N.S. | >0.05 | N.S. | >0.05 |
| PE(18:0_20:4) | [M-H]^-^ | 283.2701,  303.3125,  500.2903 | 13.64 | 766.5358  (-4.42) | 1.76 | 1.34E-05 | 1.48 | 3.80E-04 | 1.5 | 1.33E-02 |
| PE(22:6_22:0) | [M-H]^-^ | 196.1041 | 15.01 | 846.6036  (2.13) | 0.75 | 4.70E-05 | N.S. | >0.05 | N.S. | >0.05 |
| PE(P-16:0_20:4) | [M-H]^-^ | 122.1052,  285.2209,  303.2441 | 11.20 | 722.5100  (-4.12) | 0.73 | 3.50E-05 | N.S. | >0.05 | N.S. | >0.05 |
| PE(P-18:1_20:4) | [M-H]^-^ | 285.2315,  303.2607,  500.2702 | 11.54 | 748.5257  (-3.89) | 0.8 | 8.30E-04 | N.S. | >0.05 | N.S. | >0.05 |
| PE(P-18:0_18:1) | [M-H]^-^ | 281.2638,  446.3802 | 15.05 | 728.5564  (-4.94) | 0.76 | 4.74E-06 | N.S. | >0.05 | N.S. | >0.05 |

**Table S2-1.** Potential lipid biomarkers in plasma of APP/PS1 mice with different ages (N.S. denotes no significant difference).

| Potential biomarker  (possible identification） | Adduct ion | MS/MS | Retention time (min) | m/z  (mass error, ppm) | Age (month) | | | | | |
| --- | --- | --- | --- | --- | --- | --- | --- | --- | --- | --- |
|  |  |  |  |  | 2 | | 3 | | 7 | |
|  |  |  |  |  | Fold change | P value | Fold change | P value | Fold change | P value |
| PC(16:0_22:6) | [M+Acetate]^-^ | 255.1036 | 8.29 | 864.5710  (-4.99) | N.S. | >0.05 | 1.11 | 2.78E-02 | N.S. | >0.05 |
| PC(18:1_22:5) | [M+Acetate]^-^ | 281.1013 | 11.71 | 892.6022  (-4.27) | N.S. | >0.05 | 1.22 | 5.62E-03 | 1.28 | 3.59E-02 |
| PC(18:0_22:4) | [M+Formate]^-^ | 265.2703,  283.2561,  419.2448 | 14.46 | 882.6197  (-3.94) | 1.26 | 1.28E-03 | 1.48 | 5.95E-07 | 2.14 | 7.08E-06 |
| PC(P-16:0_18:3) | [M+Formate]^-^ | 277.2306 | 10.57 | 784.5479  (-2.55) | 0.56 | 3.45E-04 | 0.42 | 6.95E-07 | 0.61 | 5.77E-03 |
| PS(18:0_18:1) | [M-H]^-^ | 165.9912  265.2417,  283.2641 | 9.24 | 788.5415  (-4.06) | 2.16 | 5.09E-07 | N.S. | >0.05. | N.S. | >0.05. |
| SM(d18:0_22:1) | [M+H]^+^ | 196.1032,  282.2615,  684.4553 | 13.27 | 787.6698  (1.31) | 0.57 | 4.27E-05 | 0.44 | 2.64E-05 | N.S. | >0.05 |
| Glc/GalCer(d18:1_24:1) | [M+Na]^+^ | 163.1018,  282.0534,  444.3421 | 16.39 | 832.6600  (2.82) | 0.85 | 3.58E-02 | 0.43 | 7.55E-04 | N.S. | >0.05 |
| CE(20:5) | [M+NH_4_]^+^ | 285.2603  369.4041 | 18.52 | 688.6052  (2.10) | 0.38 | 1.14E-07 | 0.27 | 1.06E-08 | 0.53 | 2.80E-05 |
| CE(16:1) | [M+NH_4_]^+^ | 237.2108,  369.4216 | 18.98 | 640.5600  (2.71) | 0.5 | 3.52E-07 | 0.5 | 4.44E-04 | 0.56 | 9.58E-04 |
| 27,8-HDC | [M+H]^+^ | 253.1976,  271.2068,  365.3229,  383.3342,  385.2355 | 3.08 | 401.3422  (1.89) | 1.4 | 2.03E-02 | 2.9 | 6.20E-06 | N.S. | >0.05 |
| Docosahexaenoic acid | [M-H]^-^ | 283.2551 | 6.54 | 327.2333  (1.12) | 0.48 | 1.37E-03 | N.S. | >0.05 | N.S. | >0.05 |
| Arachidonic acid | [M-H]^-^ | 177.1461,  231.2603,  259.2558 | 6.76 | 303.2334  (1.44) | 0.44 | 2.86E-04 | N.S. | >0.05 | N.S. | >0.05 |
| Hydroxyoctadecanoic acid | [M-H]^-^ | 237.2336,  255.2701 | 7.70 | 299.2595  (1.10) | 0.49 | 1.38E-04 | N.S. | >0.05 | N.S. | >0.05 |
| Stearic acid | [M-H]^-^ | 239.2802,  265.2531 | 9.7 | 283.2648  (2.09) | 0.28 | 5.93E-07 | N.S. | >0.05 | N.S. | >0.05 |

**Table S2-2.** Potential lipid biomarkers in plasma of APP/PS1 mice with different ages.

| Potential biomarker  (possible identification） | Adduct ion | MS/MS | Retention time (min) | m/z  (mass error, ppm) | Age (month) | | | | | |
| --- | --- | --- | --- | --- | --- | --- | --- | --- | --- | --- |
|  |  |  |  |  | 2 | | 3 | | 7 | |
|  |  |  |  |  | Fold change | P value | Fold change | P value | Fold change | P value |
| DG(16:1_22:6) | [M+Na]^+^ | 311.2709,  385.2443 | 10.42 | 661.4808  (-2.40) | 2.31 | 1.37E-03 | 2.86 | 2.58E-06 | 1.57 | 3.83E-03 |
| DG(18:2_18:2) | [M+Na]^+^ | 337.2780 | 13.97 | 639.4980  (3.36) | 2.45 | 4.90E-05 | 4.57 | 2.41E-08 | 2.22 | 2.77E-06 |
| DG(14:0_20:2) | [M+Na]^+^ | 211.2083,  245.1169,  263.2406 | 16.27 | 615.4979  (4.20) | N.S. | >0.05 | N.S. | >0.05 | 1.53 | 1.23E-02 |
| TG(16:1_18:2_22:6) | [M+NH_4_]^+^ | 573.4938,  621.4941,  647.5111 | 18.01 | 918.7550  (0.61) | N.S. | >0.05. | 2.67 | 6.12E-07 | 1.74 | 8.41E-05 |
| TG(16:1_16:1_16:1) | [M+NH_4_]^+^ | 523.5023,  545.3218,  573.4704 | 18.28 | 818.7201  (-3.87) | N.S. | >0.05 | 3.08 | 4.49E-07 | N.S. | >0.05 |
| TG(16:1_16:1_18:2) | [M+NH_4_]^+^ | 547.4839,  573.4945,  599.5091 | 18.31 | 844.7394  (0.61) | N.S. | >0.05 | 4.1 | 1.36E-08 | 1.62 | 9.11E-05 |
| TG(16:1_18:2_20:4) | [M+Na]^+^ | 335.2616,  573.4934,  597.4941,  623.5054 | 18.62 | 899.7100  (0.07) | N.S. | >0.05 | 2.22 | 2.14E-08 | 1.39 | 4.07E-06 |
| TG(15:0_16:0_20:3) | [M+NH_4_]^+^ | 537.4638,  587.5150,  601.5320 | 18.76 | 860.7678  (-2.82) | N.S. | >0.05 | 2.38 | 9.21E-06 | 1.58 | 2.91E-05 |
| TG(16:0_18:0_18:2) | [M+NH_4_]^+^ | 575.5093  579.5328,  603.5406 | 19.10 | 876.7998  (-1.89) | N.S. | >0.05 | 2.15 | 1.83E-05 | N.S. | >0.05 |
| TG(16:0_18:2_20:1) | [M+NH_4_]^+^ | 575.5094,  605.5542,  629.5567 | 19.12 | 902.8171  (-0.04) | N.S. | >0.05 | 2.15 | 9.24E-07 | N.S. | >0.05 |

**Table S2-3.** Potential lipid biomarkers in plasma of APP/PS1 mice with different ages.

| Potential biomarker  （possible identification） | Adduct ion | MS/MS | Retention time  (min) | m/z  (mass error, ppm) | Age (month) | | | | | |
| --- | --- | --- | --- | --- | --- | --- | --- | --- | --- | --- |
|  |  |  |  |  | 2 | | 3 | | 7 | |
|  |  |  |  |  | Fold change | P value | Fold change | P value | Fold change | P value |
| 11-dehydro-TXB2 | [M-H]^-^ | 279.1602, 295.1917 | 0.78 | 367.2121  (-1.37) | 0.57 | 3.39E-03 | 0.59 | 1.29E-03 | 1.84 | 9.36E-05 |
| Hydroxy-E4-neuroprostane | [M-H]^-^ | 357.2103,  313.2213 | 1.26 | 375.2171  (-1.51) | 0.63 | 2.17E-02 | N.S. | >0.05 | 2.15 | 5.23E-06 |
| Prostaglandin D3 | [M-H]^-^ | 287.1904, 277.1802 | 1.28 | 349.2016  (-1.16) | 0.52 | 3.17E-03 | 0.63 | 2.59E-02 | 1.84 | 1.59E-04 |
| Tetracosanoic acid | [M-H]^-^ | 59.0321,  125.0273 | 8.40 | 367.3596  (3.95) | 0.65 | 1.04E-02 | 0.74 | 2.53E-02 | N.S. | >0.05 |
| Leukotriene D4 | [M+Formate]^-^ | 143.0521 | 11.05 | 541.2577  (-2.53) | 2.28 | 3.87E-05 | N.S. | >0.05 | 0.54 | 1.32E-04 |
| PE(22:5_22:6) | [M+Formate]^-^ | 196.0342,  329.2521,  526.2962 | 7.81 | 882.5260  (-3.60) | 1.3 | 3.00E-03 | N.S. | >0.05 | 1.7 | 8.53E-07 |
| PE(15:0_22:4) | [M+Formate]^-^ | 241.1250 | 9.51 | 798.5276  (-1.88) | N.S. | >0.05 | N.S. | >0.05 | 2.3 | 1.57E-07 |
| PC(14:0_16:0) | [M+H]^+^ | 184.0732,  239.2366,  265.2521,  523.8331,  647.8901 | 8.14 | 706.5405  (3.39) | 0.53 | 2.72E-08 | 0.55 | 4.43E-07 | 0.55 | 6.50E-06 |
| PC(18:0_24:1) | [M+H]^+^ | 166.0635,  184.0736,  349.4032,  504.3465,  689.6447 | 17.20 | 872.7070  (-3.81) | N.S. | >0.05 | 0.6 | 3.85E-03 | N.S. | >0.05 |
| DG(14:0_18:0) | [M+NH_4_]^+^ | 285.2702,  341.3210 | 14.22 | 569.5154  (3.47) | 0.76 | 1.68E-02 | 0.68 | 7.07E-05 | 0.55 | 4.51E-03 |
| SulfgalCer(d18:1_24:1) | [M-H]^-^ | 237.2103,  321.2342 | 12.61 | 888.6268  (3.17) | 1.46 | 1.28E-03 | N.S. | >0.05 | N.S. | >0.05 |
| Cer(d18:1_18:1) | [M+H] ^+^ | 262.2514,  280.2617,  516.5064,  528.5301,  546.5200 | 12.79 | 564.5293  (2.78) | 0.61 | 3.65E-03 | 0.85 | 8.91E-03 | 1.37 | 3.08E-05 |

**Table S3.** Potential lipid biomarkers in brain of APP/PS1 mice with different ages (N.S. denotes no significant difference).

**Reference**

1. Zhang, X. J. *et al*. Hippocampus proteomics and brain lipidomics reveal network dysfunction and lipid molecular abnormalities in APP/PS1 mouse model of Alzheimer's disease. Journal of Proteome Research, dol:10.1021/acs. jproteome.0c00255.
